# The influence of meteorological factors and terrain on air pollution concentration and migration: a geostatistical case study from Krakow, Poland

**DOI:** 10.1038/s41598-022-15160-3

**Published:** 2022-06-30

**Authors:** Tomasz Danek, Elzbieta Weglinska, Mateusz Zareba

**Affiliations:** Department of Geoinformatics and Applied Computer Science, Faculty of Geology, Geophysics and Environmental Protection, AGH University of Science and Technology, Adama Mickiewicza 30, 30-059 Kraków, Malopolska Poland

**Keywords:** Environmental sciences, Engineering, Mathematics and computing

## Abstract

Despite the very restrictive laws, Krakow is known as the city with the highest level of air pollution in Europe. It has been proven that, due to its location, air pollutants are transported to this city from neighboring municipalities. In this study, a complex geostatistical approach for spatio-temporal analysis of particulate matter (PM) concentrations was applied. For background noise reduction, data were recorded during the COVID-19 lockdown using 100 low-cost sensors and were validated based on indications from reference stations. Standardized Geographically Weighted Regression, local Moran’s I spatial autocorrelation analysis, and Getis–Ord Gi* statistic for hot-spot detection with Kernel Density Estimation maps were used. The results indicate the relation between the topography, meteorological variables, and PM concentrations. The main factors are wind speed (even if relatively low) and terrain elevation. The study of the PM2.5/PM10 ratio allowed for a detailed analysis of spatial pollution migration, including source differentiation. This research indicates that Krakow’s unfavorable location makes it prone to accumulating pollutants from its neighborhood. The main source of air pollution in the investigated period is solid fuel heating outside the city. The study shows the importance and variability of the analyzed factors’ influence on air pollution inflow and outflow from the city.

## Introduction

Air pollution has an impact on human health^1^. It has been proven that elevated concentrations of PM1, PM2.5 and PM10 may contribute to the development of diseases such as lung cancer^2^, asthma^3^, pneumonia^4^, high blood pressure^5^, Alzheimer’s and Parkinson’s disease^6^. 7% of global deaths are caused by overexposure to air pollution^7^. It is estimated that air pollution in Poland shortens life expectancy by almost 3 years, which is more than the European Union (EU) average^8^. To protect citizens from overexposure, the EU issued directive 2008/50/EC on ambient air quality and cleaner air for Europe (AAQD)^9^. Member States, including Poland, should adjust their laws to EU regulations. The air quality standards in Poland (in line with EU standards) are 40 $\upmu \mathrm{g}/\mathrm{m}^{3}$ (1-year averaged) and 50 $\upmu \mathrm{g}/\mathrm{m}^{3}$ (24-h averaged) for PM10 and 25 $\upmu \mathrm{g}/\mathrm{m}^{3}$ for PM2.5 (1-year averaged). Reference measurements can be divided into gravimetric manual measurements (norm PN-EN 12341) and automatic measurements (norm PN-EN 16450). The advantages of these measurements are their high accuracy and low uncertainties, but they are very expensive and characterized by very low spatial density (only 10 stations in the almost 15,000 $\mathrm{km}^{2}$ area around Krakow). There are also low-cost sensors (LCS) that are less accurate than reference measuring stations, have greater uncertainties, and are significantly impacted by external meteorological conditions. In contrast, LCS are characterized by a very dense spatial network^10^, which allows them to be used for advanced spatial analyses after proper data preparation. Bulot et al.^11^ confirmed that they can be applied in spatial studies in urban areas. In this study, Airly LCS were used. Their measurement correctness was very high in the examined period and was close to the reference measurements^12^. LCS uncertainties are higher than gravimetric measurements. It is also not easy to calculate them, as measurements based on light scattering can be affected by many meteorological factors^13^.

Krakow is a city with a long history of air pollution problems and has a significant history in the fight to reduce it. Kobus et al.^14^ indicated the importance of providing air quality information to cities’ residents to help make them aware of this problem. Danek and Zareba^12^ presented similar conclusions on the basis of an analysis of long-term trends and seasonality of PM10 indications in Krakow. In particular, they showed the effectiveness of social and informational campaigns, but also specific legal actions. The main sources of pollution have changed over the years. In the early 1970s, the metallurgy industry was the main source. As the city’s population grew, the share of fossil fuel heating as a source of pollution began to increase^15^ and it is now the dominant source in the winter months^16^. Surprisingly there is a total ban on solid fuel use for heating in Krakow, so the main sources of pollution are located outside the city. The official government research on PM10 composition showed that the carbon fraction has a 50% share, secondary aerosols (inorganic) have a 20% share, 10% is related to remaining ions, and the metal fraction is no more than 4%. Isotopic studies have proved that the burning of coal causes the greatest impact on the carbon fraction, but this changes depending on the time of year. In the cold period (late autumn, winter, and early spring), the main source of the carbon fraction is solid fuel heating, while in the warm period (late spring, summer, and early autumn) this is only about 20%. The second main factor is car transportation, which also varies depending on the time of year. Its concentration in the annual distribution is inversely proportional to the share of the fraction coming from solid fuel heating, which varies from 11% in winter up to 42% in summer. Natural emissions have a 30% share in the carbon fraction, and this remains constant throughout the year^17^.

The current air pollution problem in Krakow is related to this city’s geographical location^18^, but there are no detailed studies regarding this factor in combination with meteorological variables and pollutant source differentiation. This city is situated in a valley that is crossed latitudinally by the Vistula river valley. The specific morphology of the Krakow area makes vertical and horizontal natural air ventilation very difficult^19^. The Vistula river enters the city from the west, where the Oswiecimska basin and Krakow Gate are situated. It is part of fault-block hills. The river leaves the city from the east (lowland Sandomierz basin). The north upheaval is related to the occurrence of Jurassic limestones (known as the Polish Jurassic Highland). The southern upheaval is part of the Wielickie foothills and consists mostly of limestone^20^. The Tatra Mountains and the Carpathian inner-mountain basin are less than 100 km in a straight line to the south of the city, which causes the occurrence of strong, warm halny (foehn-type) winds in Krakow^21^. The air pollution problem in Krakow is critical. Despite many regulations prohibiting the use of fossil fuels for heating, pollutants still migrate to the city from external locations, making it one of the most polluted cities in the world^22^. The research shows the indisputable influence of meteorological factors on PM concentrations in the air. The impact of these factors on air pollution and its prediction^23^ varies according to many characteristic local climate variables and human activity and energy consumption^24^. Depending on the studied area, the dominant factors vary, e.g., temperature in the USA^25^, humidity with temperature in Bangladesh^26^, and air pressure in China^27^. Depending on elevation, atmospheric properties vary and can also influence PM concentrations and long-distance pollution migration^28^. The COVID-19 pandemic period provided unique conditions for geospatial observations^29^. In this case, the effects of solid fuel heating on PM concentrations with very limited background noise caused by car transportation were investigated. The typical approach that is based on reference sensors does not provide sufficient density for quantitative analyses of the influence of meteorological factors and the influence of topography. There is also no unambiguous indicator determining the origin of pollutants that can be used in time and space directly from concentration measurements without the need to perform complex radiometric analyses. This research allows this gap to be filled. The aim of this work is detailed investigation of the influence of meteorological factors and morphology on air pollution in Krakow and the migration of these pollutants using geostatistical methods, including standardized Geographically Weighted Regression (GWR), local Moran’s I spatial autocorrelation analysis, and Getis–Ord Gi* statistic for hot-spot detection. Each of the geostatistical methods used has some limitations, so multiple methods were integrated to minimize these uncertainties. Kernel density estimate (KDE) maps with box and swarm plots were analyzed to determine patterns of meteorological factors and facilitate the distribution study.

It was specifically hypothesized that it is possible (1) to investigate how the influence of meteorological factors on pollution concentration changes spatially; (2) to quantify the temporal variability of the influence of these factors; (3) to connect these changes with topography; (4) to track the sources of pollutants from solid fuel heating. The conclusions are extended with an analysis of the results based on the topography of the research area and the analysis of pollution sources using the PM2.5/PM10 ratio. This indicator was chosen because research shows that it is a good indicator of whether PM pollution is anthropogenic-related^30^ or not^31^. The presented research is unique because it uses accurate, high-resolution, short-time measurements, sampled in a regular grid in a very specific area. Most of the studies conducted so far try to show the dominant meteorological factor based on many years of measurements at a single point or a few points. In this study, the impact of all factors at many points in a short period was analyzed. In the examined period, the high variability of parameters that are indicated in the literature as dominant (temperature, pressure) is not expected. Thanks to the use of a high-resolution terrain model, it was possible to accurately determine the impact of topography on the migration of pollutants in a relatively small area of complicated morphology. This is not possible when sparse or one-point observation is conducted. Until now, this has been difficult due to the lack of a dense network of sensors or the low resolution of satellite air pollution analyses (especially in the case of large relative elevation changes within a short distance). The impact of background noise was significantly reduced due to limited car traffic during the COVID-19 pandemic. An unusual approach to the analysis of the PM2.5/PM10 ratio is also presented to distinguish different anthropogenic dust sources (typically used to analyze the origin of PMs from natural and anthropogenic sources). In a spatio-temporal sense, this is one of the most detailed studies conducted so far on air pollutants generated by solid fuel heating.

## Methods

### Data source and validation

**Figure 1:**
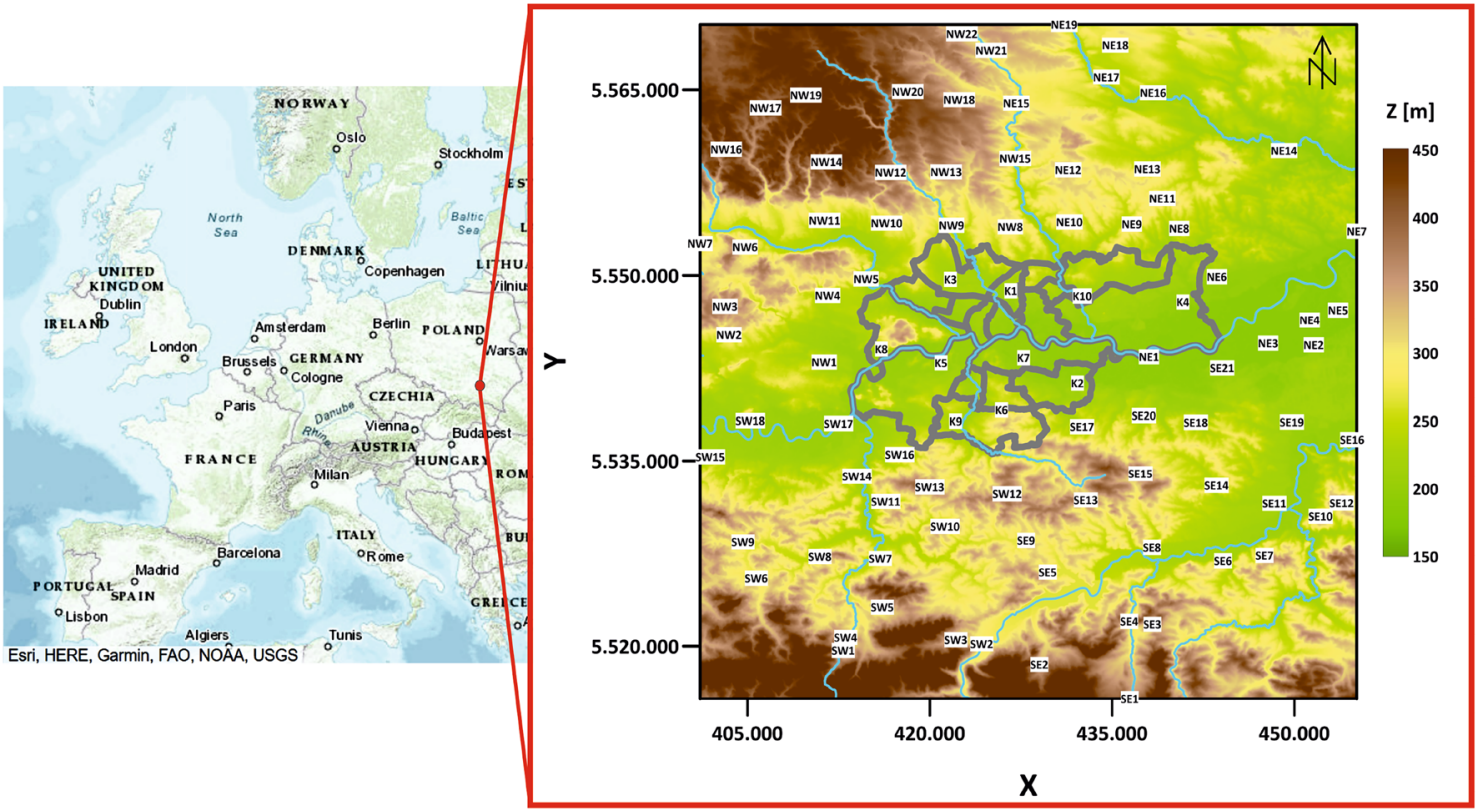
Krakow topography map (digital terrain model) with Airly sensor locations and their IDs (white rectangle), together with the borders of Krakow districts (grey lines and the main rivers (blue lines). Digital terrain model source: European Union, Copernicus Land Monitoring Service 2022, European Environment Agency (EEA).

1-hour averaged measurements from 90 LCS stations located in Krakow and its surroundings were used. Figure 1 shows the locations of these stations and the digital terrain model map (source: European Union, Copernicus Land Monitoring Service 2022, European Environment Agency (EEA)). Sensors were divided into 5 groups (due to their geographic location):

1. **K** group—sensors located in Krakow urban area (Table 1);
2. **NW** group—sensors located in the north-west section outside the Krakow urban area (Table 2);
3. **SW** group—sensors located in the south-west section outside the Krakow urban area (Table 3);
4. **NE** group—sensors located in the north-east section outside the Krakow urban area (Table 4);
5. **SE** group—sensors located in the south-east section outside the Krakow urban area (Table 5).

The data comes from the Airly sensors network (https://map.airly.org/) and were downloaded using free data access from the Airly API. Sensors used in this study are optical-type detectors that use the light scattering phenomena for PM measurement, but this can be influenced by many factors^32^. The measured quantities from the Airly API are already verified and calibrated with the use of machine learning algorithms and indications from reference stations. It should be borne in mind that despite the proven high accuracy of Airly LCS measurements, it is important to compare them with reference measurements as these are characterized by the lowest measurement uncertainties and the highest accuracy of indications^13^. Validation of the indications of these sensors (including the intervals studied in this paper) has been the subject of many studies^12^. Research shows that the Plantower 5003 sensor (used by Airly) provides a measurement accuracy that may be lower at high relative humidity. Despite the greater accuracy of LCS measurements for PM10, the indications of this parameter may differ significantly from the reference indications (this is not the case for PM2.5). This is related to the dominant particle-type changes within the PM10 fraction. The greatest discrepancies occur for sensors affected by street dust resuspension and in areas where building construction and demolition are occurring. In this study, the COVID-19 pandemic and partial lockdown significantly reduced the impact of these factors^33^.

Bartyzel et al.^34^ performed an analysis of the compliance of Airly sensors with a reference station according to the PN-EN 16450:2017-05 standard. These authors showed that proper calibration significantly reduces measurement uncertainty and that the accuracy increases with the average daily concentration. Adamiec et al.^35^ showed that thanks to the use of dedicated calibration techniques and appropriate validation of manufacturers’ indications, Airly sensors can complement reference measurements. Research carried out in Krakow with the use of these sensors also shows their high accuracy and compliance with the indications of a reference station, both on windless days^12^ and during periods with strong winds^22^.

For the analysis, in order to focus on solid fuel heating-related pollution, we wanted to select days during the COVID-19 pandemic period when car traffic density was up to 40% lower compared to previous years. The second motivation was the selection of days on which the wind was relatively light to limit the effect of wind as a factor in the models. These requirements were met on March 11th and 18th, 2021. On March 11th, analyzing the hours of 00:00, 04:00, and 08:00, the process of pollutants moving away from the city is visible, while on March 18th at 12:00, 18:00, and 24:00, an inflow of pollutants can be observed (compare Danek and Zareba’s study^12^).

**Table 1:** Sensor location (district) and elevation in Krakow.

LCS ID	LCS elevation (m)	Location (Krakow district)
K1	216	Pradnik Czerwony
K2	199	Biezanow Prokocim
K3	224	Pradnik Bialy
K4	201	Nowa Huta
K5	212	Debniki
K6	235	Podgorze Duchackie
K7	200	Podgorze
K8	228	Zwierzyniec
K9	235	Swoszowice
K10	208	Wzgorza Krzeslawickie

**Table 2:** Sensor location and elevation in the north-west section (outside the urban areas of Krakow).

LCS ID	LCS elevation (m)	Location
NW1	249	Liszki
NW2	277	Rybna
NW3	374	Sanka
NW4	241	Aleksandrowice
NW5	220	Szczyglice
NW6	317	Nawojowa Gora
NW7	274	Tenczynek
NW8	280	Bolen
NW9	243	Trojanowice
NW10	266	Tomaszowice
NW11	269	Wieckowice
NW12	388	Bialy Kosciol
NW13	372	Brzozowka
NW14	424	Bedkowice
NW15	253	Wilczkowice
NW16	421	Paczoltowice
NW17	389	Czubrowice
NW18	334	Przybyslawice
NW19	446	Gotkowice
NW20	416	Skala
NW21	297	Grzegorzowice Wielkie
NW22	324	Golyszyn

**Table 3:** Sensor location and elevation in the south-west section (outside the urban areas of Krakow).

LCS ID	LCS elevation (m)	Location
SW1	328	Harbutowice
SW2	291	Myslenice
SW3	329	Myslenice II
SW4	327	Sulkowice
SW5	339	Rudnik
SW6	282	Brody
SW7	252	Krzywaczka
SW8	292	Zarzyce Male
SW9	308	Stanislaw Dolny
SW10	340	Wlosan
SW11	237	Radziszow
SW12	350	Rzeszotary
SW13	391	Mogilany
SW14	225	Rzozow
SW15	236	Brzeznica
SW16	279	Skawina
SW17	209	Jeziorzany
SW18	217	Czernichow

**Table 4:** Sensor location and elevation in the north-east section (outside the urban areas of Krakow).

LCS ID	LCS elevation (m)	Location
NE1	193	Grabie
NE2	184	Zabierzow Bochenski II
NE3	188	Wola Batorska
NE4	187	Zabierzow Bochenski
NE5	185	Chobot
NE6	203	Tropiszow
NE7	201	Nowe Brzesko
NE8	246	Karniow
NE9	289	Luborzyca
NE10	285	Prawda
NE11	306	Pietrzejowice
NE12	316	Zagorzyce Stare
NE13	252	Luborzyca II
NE14	221	Proszowice
NE15	277	Iwanowice
NE16	229	Waganowice
NE17	258	Slomniki
NE18	264	Prandocin
NE19	272	Smrokow

**Table 5:** Sensor location and elevation in the south-east section (outside the urban areas of Krakow).

LCS ID	LCS elevation [m]	Location
SE1	350	Wisniowa
SE2	343	Trzemesnia
SE3	340	Kwapinka
SE4	275	Czaslaw
SE5	289	Zakliczyn
SE6	272	Zreczyce
SE7	316	Jaroszowka
SE8	236	Winiary
SE9	277	Czechowka
SE10	296	Buczyna
SE11	214	Pierzchow
SE12	323	Dabrowica
SE13	364	Raciborsko
SE14	230	Wiatowice
SE15	402	Sulow
SE16	200	Cikowice
SE17	258	Wieliczka
SE18	224	Zagorze
SE19	212	Klaj
SE20	230	Przebieczany
SE21	202	Niepolomice

### Geostatistical methods

Statistical analyses were performed with the use of *ArcGIS Pro*^36^ and *Python*^37^ (including libraries such as seaborn^38^ and scikit-learn^39^). GWR, Moran’s *I* spatial autocorrelation analysis, and Getis–Ord $G_{i}^{*}$ hot-spot detection were performed in *ArcGIS Pro* software^36^ for all sensors presented in Tables 1, 2, 3, 4 and 5. To be able to assess the importance of individual meteorological factors, the data were standardized before performing GWR^40^ using z-score according to Eq. (1):

1 $$\begin{aligned} z = \frac{x-\mu }{\sigma } \end{aligned}$$

where *x* is original sample value, $\mu$ is simple mean of all observations, $\sigma$ is standard deviation.

The multivariate kernel density estimations were calculated for each meteorological factor to study the average relationship between them and the PM concentrations. Analyses of descriptive statistics and patterns were also performed. Exploratory data analysis (EDA) includes box and swarm plots. Box plots provide very similar information to KDEs but in a simplified form, while swarm plots help in understanding data structure.

#### Geographically Weighted Regression

Geographically weighted regression (GWR)^41^ is a local form of regression that is used to model spatially varying relationships. In *ArcGIS Pro* software^36^, this function is realized using a Geographically Weighted Regression tool that provides three types of regression models: continuous (Gaussian), binary (Logistic) and count (Poisson). The type of model to analyze should be chosen based on how the dependent variable is measured. The next important assumption to make is choosing the neighborhood type and the neighbor selection method. In the GWR method, N local linear regression equations are calculated using a certain distance-based weighting scheme. To determine the area from which the model should investigate spatial variation, crossvalidation is performed^42^.

The GWR model can be expressed by Eq. (2):

2 $$\begin{aligned} y_{i}=\sum _{j=0}^{M}\beta _{j}(u_{i},v_{i})x_{ij}+\varepsilon _{i} \end{aligned}$$

for $i=1,\ldots ,N$ and $j=0,\ldots ,M$, where $(u_{i},v_{i})$ gives the point of the coordinates’ region *i*, $y_{i}$ is the dependent variable, $\beta _{j}$ are regression coefficients, $x_{ij}$ is the *j*th variable at observation *i*, and $\varepsilon _{i}$ is the residual variable. Parameter estimation of local regression models is performed using explanatory variables derived mainly from neighboring observations. GWR detects spatial variation in model dependencies, which allows the creation of maps to explore spatial nonstationarity^41^. The GWR method has an advantage over other regression methods (e.g., Ordinary Least Square) because it does not assume a constant variance and allows for more accurate analysis when nonstationarity is present^43^. The limitations of this method are considered to be multicollinearity in local coefficients^44^ and computationally demanding cross-validation for large datasets^42^. Despite some limitations, GWR is a valuable technique for studying spatial nonstationarity^45^.

#### Local Moran’s *I*

Local Moran’s *I* is a local spatial autocorrelation statistic proposed by Anselin^46^ as a way to identify local clusters and outliers. In *ArcGIS Pro* software^36^, this function is realized by the Cluster and Outlier Analysis (Anselin Local Moran’s *I*) tool. The local Moran’s *I* is given as Eq. (3):

3 $$\begin{aligned} I_{i}=\frac{x_{i}-\bar{X}}{S_{i}^{2}}\sum _{j=1,j\ne i}^{n}w_{ij}(x_{j}-\bar{X}) \end{aligned}$$

where $x_{i}$ is the value of the attribute x at location i, $\bar{X}$ is the mean of the attribute at each of n points, $x_{j}$ is the attribute value at all other locations ($j\ne i$), and $S_{i}^{2}$ is the variance of the variable *x*. The matrix of weights $w_{ij}$ defines the distance between objects. The matrix was calculated using the inverse distance method.

A positive index *I* value indicates that the study location has similar high or low values to its neighbors. This tool allows for the determination of high-high clusters (high values in a high-value neighborhood) and low-low clusters (low values in a low-value neighborhood). A negative index *I* value indicates that the study location is a spatial outlier. This tool determines high-low (a high value in a low-value neighborhood) and low-high (a low value in a high-value neighborhood) outliers. The obvious advantage of Moran’s method is its simplicity, but like any statistical method it has some limitations. The results of using local Moran’s I statistic to detect PM hot-spots are affected by the choice of the weight matrix. There is no general rule regarding when the different types of weights should be used^47^

#### Getis–Ord $G_{i}^{*}$

The Getis–Ord $G_{i}^{*}$ local statistic^48^ allows detection of local concentrations of high and low values in neighboring sites, and it tests the statistical significance of this relationship. This statistic can identify hot-spots (clusters of high attribute levels) and cold-spots (clusters of low attribute levels) with varying levels of significance. In *ArcGIS Pro* software^36^, this function is realized by Getis–Ord $G_{i}^{*}$ (High/Low Clustering). The Getis–Ord local statistic is given as Eq. (4):

4 $$\begin{aligned} G_{i}^{*}=\frac{\sum _{j=1}^{n}w_{ij}x_{j}-\bar{X}\sum _{j=1}^{n}w_{ij}}{S\sqrt{\frac{n\sum _{j=1}^{n}w_{ij}^{2}-(\sum _{j=1}^{n}w_{ij})^{2}}{n-1}}} \end{aligned}$$

High values of the $G_{i}^{*}$ index indicate objects with high PM concentration values, while low values indicate objects with low values. When the values are close to the expected value, the distribution of the analyzed attribute is random in space. $G_{i}^{*}$ statistics, similar to Moran’s technique, requires establishing a conceptualization of spatial relationships, which can result in ambiguous solutions. The Getis–Ord $G_{i}^{*}$ method is widely used and favored over other available statistics (e.g., $G_{i}$^48^, Geary’s *C*^49^, Moran’s *I*). One of the many advantages of the method mentioned by^50^ is the possibility to identify areas with increased values of the examined parameter, even if the values do not differ from the global average.

## Results

### Exploratory data analysis

**Figure 2:**
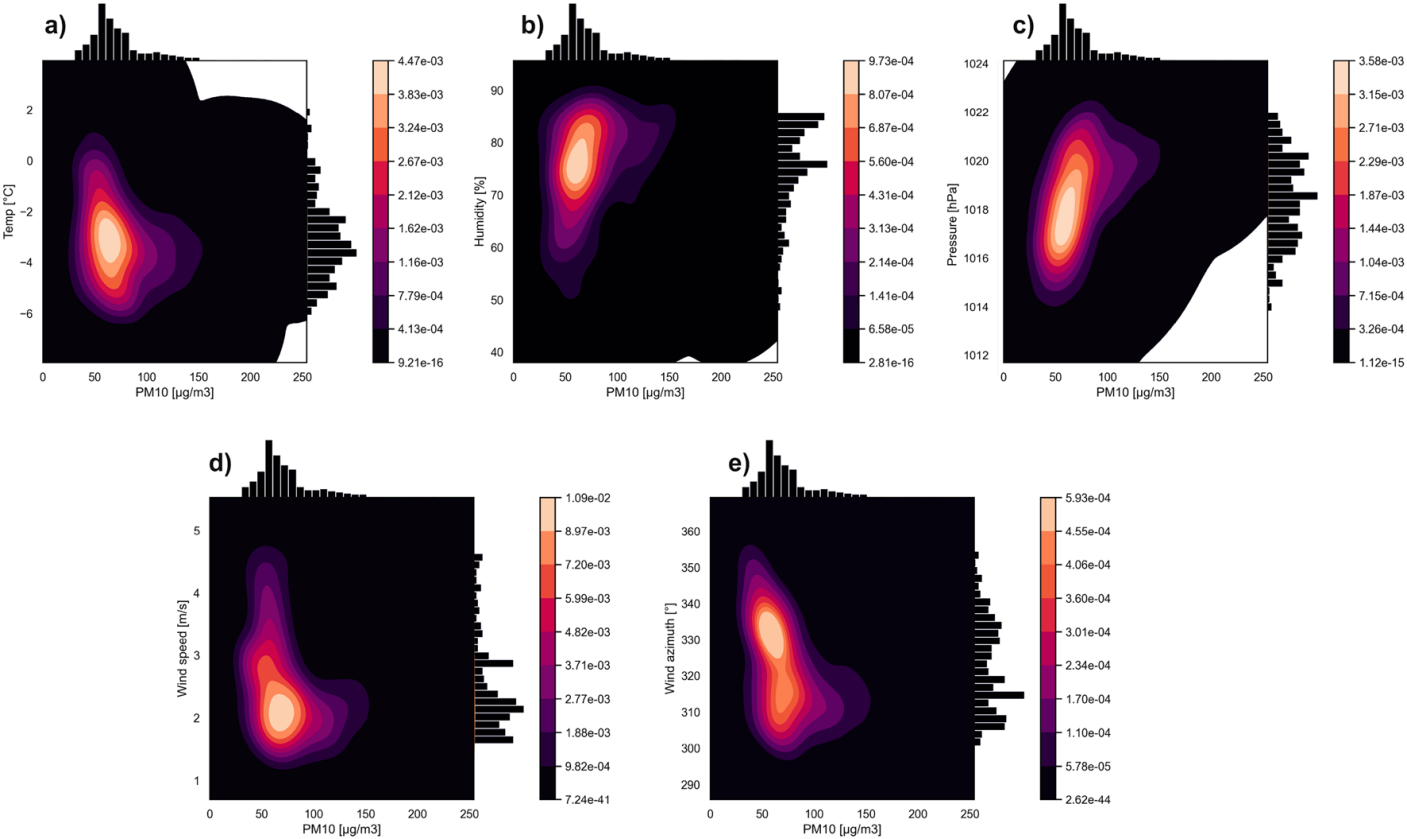
Multivariate kernel density estimations of PM10 and temperature (**a**), humidity (**b**), pressure (**c**), wind speed (**d**), wind azimuth (**e**) on the 11th of March.

**Figure 3:**
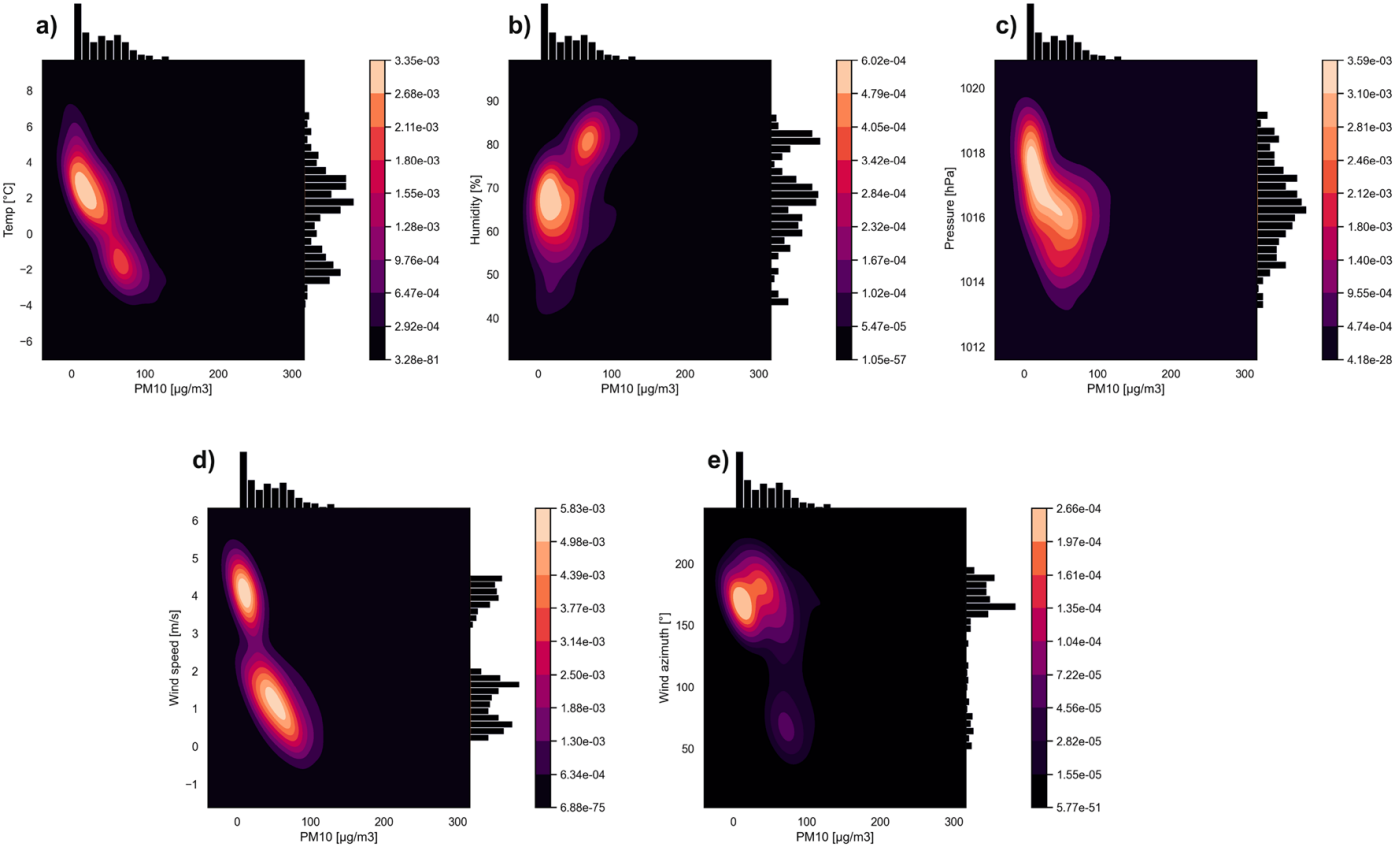
Multivariate kernel density estimations of PM10 and temperature (**a**), humidity (**b**), pressure (**c**), wind speed (**d**), wind azimuth (**e**) on the 18th of March.

Figures 2 and 3 show the multivariate kernel density estimations of PM10 and different meteorological factors on 11th and 18th March. It is clearly visible that higher PM10 values are related to lower temperatures. PM10 concentration above 50 $\upmu \mathrm{g}/\mathrm{m}^{3}$ occurred when the temperature dropped below $0\,^{\circ }\mathrm{C}$ degrees. A rapid PM10 increase is observed for the range of − 3 to − $5\,^{\circ }\mathrm{C}$ degrees (up to 150 $\upmu \mathrm{g}/\mathrm{m}^{3}$). It is important to note that on 18th March the temperature (Fig. 3a) was above $0\,^{\circ }\mathrm{C}$ degrees for some time, and the PM10 concentration was low—about 25 $\upmu \mathrm{g}/\mathrm{m}^{3}$ for the temperature range 2–4 $^{\circ }\mathrm{C}$.

In the case of relative humidity, it is clearly noticeable that values above 80% are associated with higher PM10 values. On March 18th (Fig. 3b), when the relative humidity was in the 60–70% range, the PM10 values were significantly lower than those of the 80% range. On March 11th (Fig. 2b), the humidity remained at the level of 75–85% and was positively correlated with increased concentrations of PM10. Atmospheric pressure does not display an unequivocal relationship. On March 11th (Fig. 2c) it was also possible to observe an increase in pollutants along with an increase in atmospheric pressure from 1016 to 1022 hPa, while on March 18th (Fig. 3c) this relationship was reversed. For the value of 1017–1020 hPa, PM10 pollution was negligible, but when the pressure dropped to 1014 hPa, an increase in air pollution was visible. The wind speed shows the expected trend. As wind speed decreases, air pollution increases. The winds on March 11th and 18th were relatively weak. On March 11th (Fig. 2d), the average speed was 2 m/s, while on 18th March (Fig. 3d) the two dominant speed values were 1 m/s and 4 m/s. Wind speed as low as 4 m/s is associated with nearly zero PM10 values. As for the wind azimuth, on March 11th (Fig. 2e), NW, NNW, and N winds prevailed. The highest concentration is observed for around $310\,^ {\circ }$. On March 18th (Fig. 3e), the main wind azimuths are SSW, S and SSE. The lowest concentration values are observed for the SE wind azimuth.

**Figure 4:**
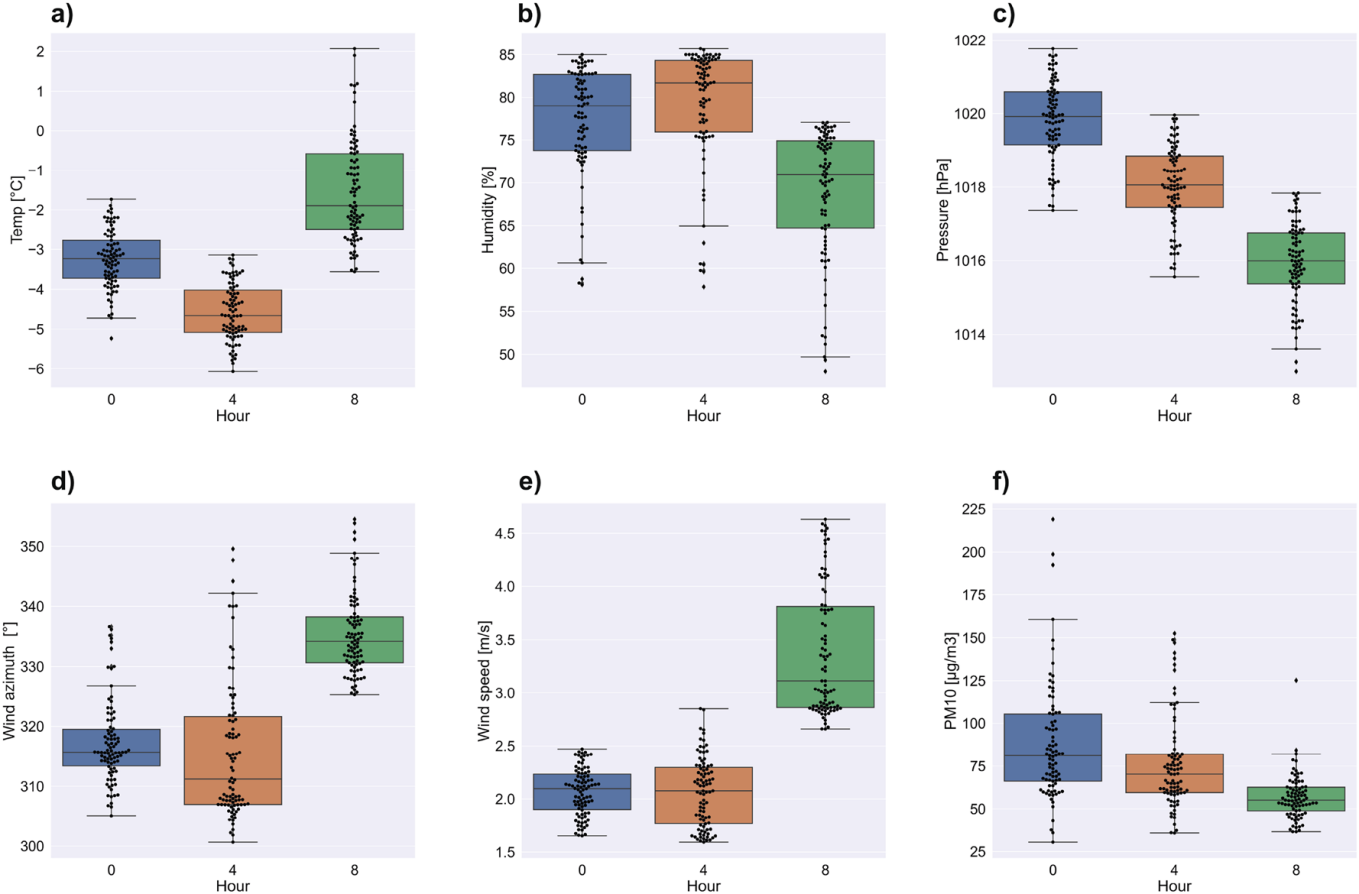
Box and swarm plots for temperature (**a**), humidity (**b**), pressure (**c**), wind azimuth (**d**), wind speed (**e**), and PM10 concentration (**f**) on the 11th of March.

The Fig. 4a–f show swarm and box plots for individual meteorological factors and PM10 indicators on March 11th at midnight, 4, and 8 am. The temperature (Fig. 4a) distribution changes with time. At midnight, a one-modal distribution is visible, while for 08:00 a three-modal distribution is present, with one mode being close to maximum. The most compact and symmetrical distribution is for midnight with the median at around $-\,3\,^{\circ }\mathrm{C}$ degrees, for 04:00 the median ($-\,4.5\,^{\circ }\mathrm{C}$ degrees) is closer to the 1st quartile. The most asymmetric distribution is for hour 08:00. The Median is around $-\,2\,^{\circ }\mathrm{C}$ degrees, but there is a very large percentage of indications where the temperature was above zero. The distributions for humidity (Fig. 4b) are asymmetrical for all hours and are multi-modal. The Median changes from 80% at midnight to 83% at 04:00 and then decreases to 70% at 08:00, The pressure (Fig. 4c) is characterized by the symmetrical and constant nature of the distribution, despite the clearly decreasing trend with successive hours. The width of the boxes and the location of the median at their centers are similar. Wind azimuth and speed distributions (Fig. 4d,e) are asymmetric. Most of the outliers are located near the maximum values, and the median is close to the first quartile. The distribution for box 3 in Fig. 4d is different. In roughly half of the boxes there is a clear multi-modal pattern with a wide box. The distributions of PM10 concentrations (Fig. 4f) are interesting. In the following hours, the width of the boxes decreases significantly. At 08:00, a very compact, basically one-modal distribution is visible with values strongly clustered around the median that amount to just over 50 $\upmu \mathrm{g}/\mathrm{m}^{3}$.

**Figure 5:**
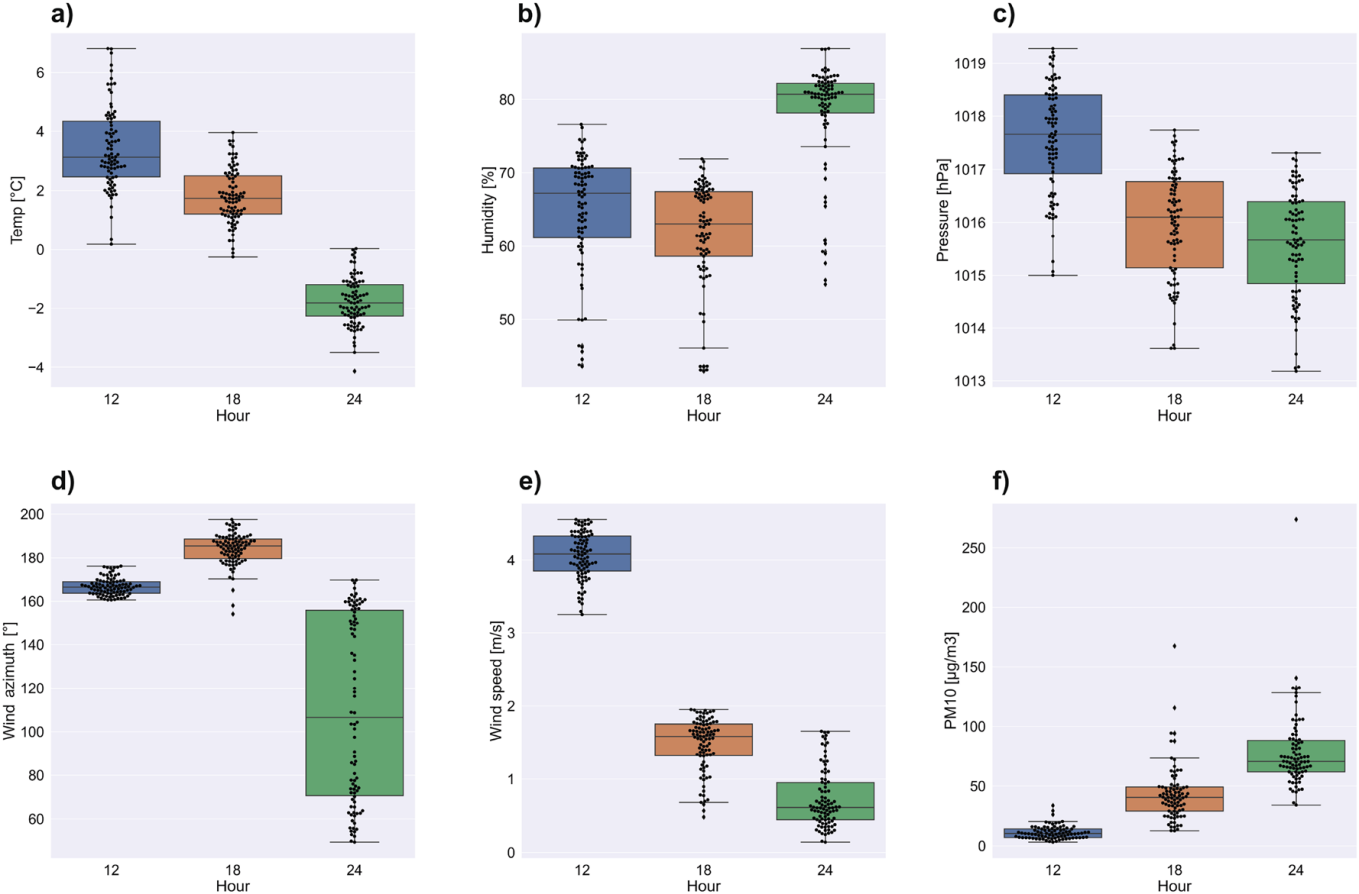
Box and swarm plots for temperature (**a**), humidity (**b**), pressure (**c**), wind azimuth (**d**), wind speed (**e**), and PM10 concentration (**f**) on the 18th of March.

Figure 5a–f show swarm and box plots for individual meteorological factors and PM10 indicators on March 18th at 12:00, 18:00, and 24:00. The temperature distribution is three-modal for 12:00 and 18:00, and two-modal for 24:00 (Fig. 5a). The most compact and symmetrical distribution is for midnight, with the median around $-2\,^{\circ }\mathrm{C}$; for 12:00, the median ($3\,^{\circ }\mathrm{C}$) is closer to the 1st quartile. In general, the temperature decreases with time. The distributions for humidity (Fig. 5b) are asymmetrical for all hours and are multi-modal (similar to those from 11th March). The Median changes from 67% at 12:00 to 63% at 18:00, and then increases up to 80% at midnight. The pressure (Fig. 5c) is characterized by quite symmetrical distributions. In contrast to 11th March, the boxes are wide and the distributions are not so compact. The distributions for wind azimuth and speed do not show a trend. It can be noticed that the wind azimuth (Fig. 5d) at 12:00 and 18:00 was the same for almost all observation points, while at midnight an extremely wide box with a multi-modal pattern is present. The highest values for wind speed (Fig. 5e) are observed for 12:00, with a compact and symmetrical distribution. For hours 18:00 and 24:00, the distributions are still compact but asymmetry is visible. At 18:00, the median is closer to the 3rd quartile with a long 1st whisker, while at midnight it is the opposite. The distribution of PM10 concentration (Fig. 5f) at 12:00 is very compact with an extremely narrow box. This situation changes over time: the distributions at each subsequent hour become less consistent with the observed shifts of the medians towards higher concentrations and with numerous outliers towards the maximum values.

### Spatial autocorrelation

#### Local Moran’s *I*

To estimate the spatial autocorrelation for PM2.5 and PM10 indicators at all sensors, the local Moran’s *I* and Getis–Ord $Gi^{*}$ were calculated. Statistical significance was assumed at the 95 percent confidence level. The inverse distance method was used for the conceptualization of the spatial relationship.

**Figure 6:**
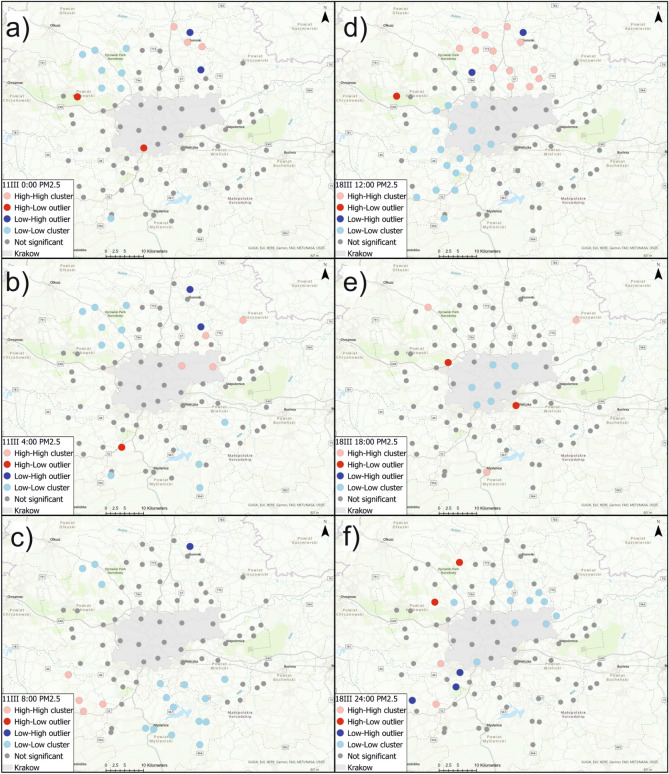
Local Moran’s I cluster maps showing high-high, low-low, low-high, and high-low spatial associations for PM2.5 concentration on March 11th and March 18th.

Figure 6a–c show local Moran’s *I* cluster maps for PM2.5 concentration on March 11th at 0:00, 4:00, and 8:00; Fig. 6d–f show local Moran’s *I* cluster maps for PM2.5 concentration on March 18th, at 12:00, 18:00, and 24:00. The local Moran’s *II* analyses identified areas of positive autocorrelation (high-high and low-low clusters) as well as areas of negative autocorrelation (high-low and low-high outliers). For PM2.5 concentration, high-high clusters were identified on March 11th at 0:00 in the north-east section (Slomniki, Waganowice, Smrokow), at 4:00 in Krakow (Wzgorza Krzeslawickie, Nowa Huta) and in the north-east (Pietrzejowice and Proszowice). At 8:00 that same day, high-high clusters of PM2.5 were identified in the south-west section (Brzeznica, Stanislaw Dolny, Brody and Zarzyce Male). On March 11th, low-low clusters were identified at 0:00 at one sensor in the S-W section (Harbutowice) and in the north-west section (Czubrowice, Gotkowice, Skala, Bedkowice, Bialy Kosciol, Wieckowice and Tomaszowice), at 4:00 south of Krakow (Wiatowice, Harbutowice, Czaslaw, Wisniowa) and at the same sensors from the N-W section for 0:00, at 8:00 south of Krakow (Rzeszotary, Raciborsko, Wiatowice, Czechowka, Winiary, Zakliczyn, Czaslaw, Kwapinka, Myslenice, Myslenice II, Trzemesnia and Wisniowa) and at three sensors located in the north-west part (Czubrowice, Gotkowice, Bedkowice). Sensors which detected anomalously high PM2.5 concentrations were Swoszowice and Nawojowa Gora at 0:00, and Krzywaczka at 4:00. Areas with anomalously low PM2.5 values in relation to neighboring areas were identified at 0:00 and 4:00 in Prandocin and Luborzyca II. At 8:00, a low-high outlier was identified only in Prandocin.

On March 18th, high-high clusters were recognized at 12:00 in the N–W (Golyszyn, Grzegorzowice Wielkie, Wilczkowice) and N–E (Skala, Przybyslawice, Iwanowice, Zagorzyce Stare,Luborzyca, Luborzyca II, Pietrzejowice, Prawda) sections, at 18:00 in Gotkowice, Proszowice and Myslenice, at 24:00 in Jeziorzany and Zarzyce Male. Low-low clusters of PM2.5 concentration were recognized at 12:00 in Krakow (Pradnik Bialy, Zwierzyniec, Debniki, Swoszowice, Podgorze Duchackie) and west of Krakow (Szczyglice, Aleksandrowice, Liszki, Czernichow, Jeziorzany, Rzozow, Skawina, Mogilany, Radziszow, Krzywaczka, Zarzyce Male, Harbutowice and Brody), at 18:00 in Krakow (Pradnik Czerwony, Wzgorza Krzeslawickie, Podgorze, Podgorze Duchackie, Swoszowice and Debniki), at 24:00 in Krakow (Swoszowice, Wzgorza Krzeslawickie and Nowa Huta) and in sensors north of Krakow (Tropiszow, Karniow, Pietrzejowice, Luborzyca, Prawda, Zagorzyce Stare, Wilczkowice and Tomaszowice). PM2.5 indications with increased values relative to neighboring sensors were detected by the sensors at Tenczynek (at 12:00), in Szczyglice and Wieliczka (at 18:00), and in Wieckowice at 24:00. Low-high outliers for PM2.5 concentration were detected at 12:00 in Brzozowka and Prandocin, and at 24:00 in Stanislaw Dolny, Radziszow and Skawina.

**Figure 7:**
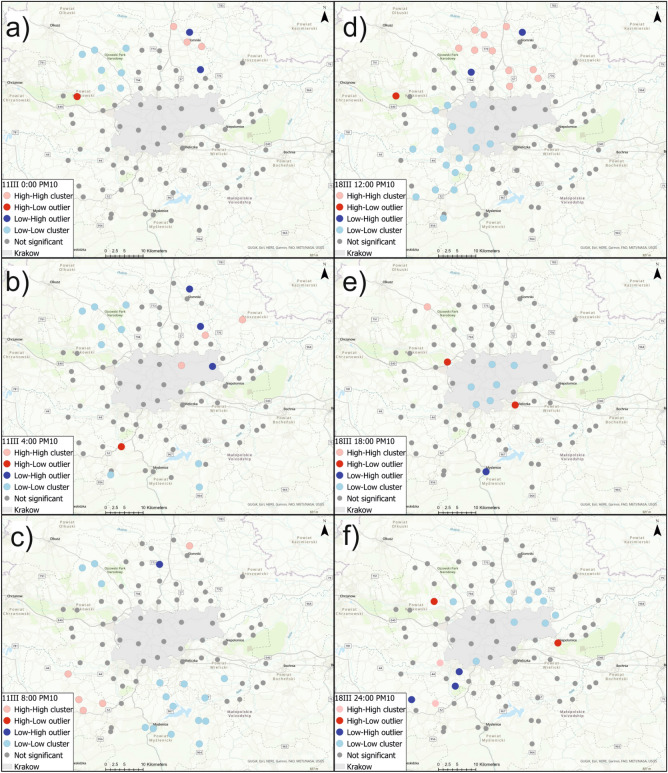
Local Moran’s *I* cluster maps showing high-high, low-low, low-high, and high-low spatial associations for PM10 concentration on March 11th and March 18th.

Figure  7a–c show local Moran’s *I* cluster maps for PM10 concentration on March 11th at 0:00, 4:00, and 8:00. Figure  7d–f show local Moran’s *I* cluster maps for PM10 concentration on March 18th at 12:00, 18:00, and 24:00. This tool allowed the selection of sensors that had high PM10 values against which there were also high PM10 indications. High-high clusters of PM10 indications were identified on March 11th at 0:00 at the same sensors as for PM2.5. On the same day at 4:00, high-value clusters of PM10 were determined in Wzgorza Krzeslawickie, Pietrzejowice, and Proszowice. On March 11th at 8:00, high-high clusters were identified in the south-west section (Brzeznica, Stanislaw Dolny, Brody, Zarzyce Male) and at one sensor to the north of Krakow (Prandocin). Local Moran’s *I* statistic made it possible to determine clusters of low values on March 11th at 0:00 at sensors located to the north-west of Krakow (Czubrowice, Gotkowice, Skala, Bedkowice, Bialy Kosciol, Wieckowice, Tomaszowice). Spatial autocorrelation analysis of PM10 at 4:00 made it possible to identify clusters including the same sensors as at 0:00, except Tomaszowice, and also to identify low-low clusters in Harbutowice, Czaslaw, and Wisniowa. On the same day at 8:00, clusters of low values of PM10 were identified southeast of Krakow (Raciborsko, Wiatowice, Winiary, Czechowka, Zakliczyn, Czaslaw, Kwapinka, Trzemesnia and Wisniowa), in the south-west section (Rzeszotary, Myslenice, Myslenice II), and northwest of Krakow (Czubrowice, Gotkowice, Bedkowice). High-low outliers were detected on March 11th only at 0:00 (Nawojowa Gora) and at 4:00 (Krzywaczka). Low-high outliers were identified at 0:00 (Prandocin and Luborzyca II), at 4:00 (Prandocin, Luborzyca II and Nowa Huta), at 8:00 in Iwanowice.

On March 18th high-high clusters were recognized at 12:00 in the north-east section (Smrokow, Iwanowice, Zagorzyce Stare, Prawda, Luborzyca II and Pietrzejowice) as well as in the north-west (Golyszyn, Grzegorzowice Wielkie, Skala, Przybyslawice), at 18:00 in Gotkowice, and at 24:00 in Jeziorzany and Zarzyce Male. Low-low clusters were identified at 12:00 in Krakow (Pradnik Bialy, Zwierzyniec, Debniki,Swoszowice, Podgorze Duchackie), in the N-W (Szczyglice, Aleksandrowice, Liszki), and in the S–W (Jeziorzany, Czernichow, Rzozow, Skawina, Radziszow, Mogilany, Brody, Zarzyce Male, Krzywaczka and Harbutowice) sections. At 18:00, low-low clusters of PM10 were detected in Krakow (Pradnik Czerwony, Wzgorza Krzeslawickie, Podgorze, Debniki, Swoszowice and Podgorze Duchackie). At 24:00, clusters of low values were identified in Krakow (Swoszowice, Wzgorza Krzeslawickie, Nowa Huta) and north of Krakow (Tropiszow, Karniow, Pietrzejowice, Luborzyca, Prawda, Zagorzyce Stare and Tomaszowice). High-low outliers were recognized at 12 in Tenczynek, at 18:00 in Szczyglice and Wieliczka, at 24:00 in Wieckowice and Niepolomice. Low-high outliers of PM10 were recognized at 12 in Brzozowka and Prandocin, at 18:00 in Myslenice, at 24:00 in Skawina, Radziszow, Stanislaw Dolny.

#### Getis–Ord $G_{i}^{*}$

**Figure 8:**
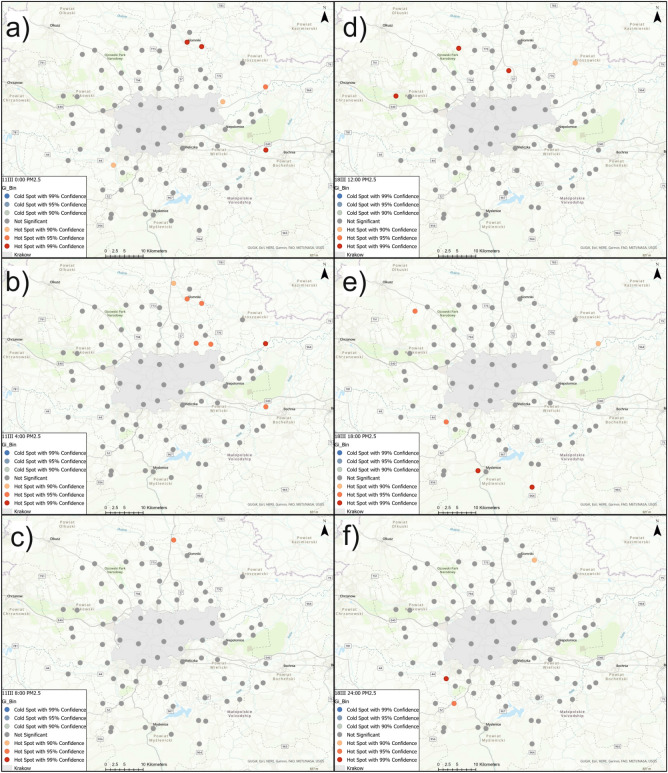
Hot-spots and cold-spots maps for PM2.5 concentration on March 11th and March 18th using Getis–Ord $G_{i}^{*}$.

**Figure 9:**
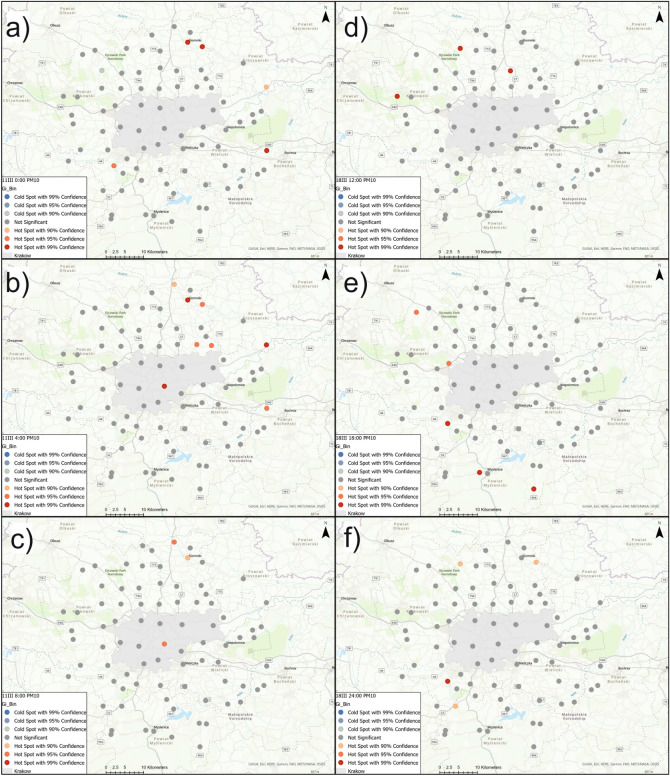
Hot-spots and cold-spots maps for PM10 con centration on March 11th and March 18th using Getis–Ord $G_{i}^{*}$.

Figure 8a–c show hot-spot and cold-spot maps for PM2.5 concentration using Getis–Ord $G_{i}^{*}$ with significance on March 11th at 0:00, 4:00, and 8:00 and Fig. 8d–f on March 18th at 12:00, 18:00, and 24:00. These local statistics did not determine any cold-spots of PM2.5 with 90, 95 or 99% confidence levels. On March 11th at 0:00, hot-spots were identified with 99% confidence in Slomniki, Waganowice, Cikowice, with 95% in Nowe Brzesko, and with 90% in Tropiszow and Rzozow. Four hours later, values with increased concentration of PM2.5 were recorded in Nowe Brzesko (99% confidence), in Slomniki, Waganowice, Luborzyca, Karniow and Cikowice (95%), and in Smrokow (90%). At 8:00, hot-spots were identified at fewer sensors: in Brody (with 99% confidence), in Smrokow (95%), and in Stanislaw Dolny (90%). On March 18th at 12:00, hot-spots of PM2.5 were determined at 4 sensors located north of Krakow: Tenczynek, Skala, Zagorzyce Stare (99%), and in Proszowice (90%). The use of local Getis–Ord $G_{i}^{*}$ statistics enabled the determination of hot-spots with 99% confidence in Myslenice and Wisniowa, with 95% in Rzozow and Czubrowice, and with 90% in Nowe Brzesko. On March 18th at 24:00, hot-spots were identified in Rzozow (99%), in Krzywaczka (95%), and in Brody and Waganowice (90%). Figure 9a–c show hot-spot and cold- spot maps for PM10 concentration using Getis–Ord $G_{i}^{*}$ with significance on March 11th at 0:00, 4:00, and 8:00 and Fig. 9d–f on March 18th at 12:00, 18:00, and 24:00. This tool did not determine any cold-spots of PM10 with 90, 95 or 99% confidence level, as in the case of PM2.5. On March 11th at 0:00, hot-spots of PM10 were found with 99% confidence in Slomniki, Waganowice and Cikowice, with 95% in Rzozow, and with 90% in Nowe Brzesko. At 4:00, hot-spots were identified in Slomniki, Nowe Brzesko and Podgorze (99%), in Waganowice, Luborzyca, Karniow, Cikowice (95%), and in Smrokow (90%). Four hours later, only a few hot-spots were found : in Brody (99%), in Smrokow, Podgorze (95%), in Slomniki and Stanislaw Dolny (90%). On March 18th at 12:00, hot-spots were identified at 3 sensors north of Krakow : in Tenczynek, Skala, and Zagorzyce Stare (99%). At 18:00, hot-spots of PM10 were identified south of Krakow in Rzozow, Myslenice II, Wisniowa (99%), and northwest of Krakow in Czubrowice and Szczyglice (95%). That same day at 24:00, one hot-spot in Rzozow (99%), one hot-spot in Brody (95%), and three hot-spots in Skala, Waganowice and Krzywaczka (90%) were identified.

### Geographically weighted regression

**Figure 10:**
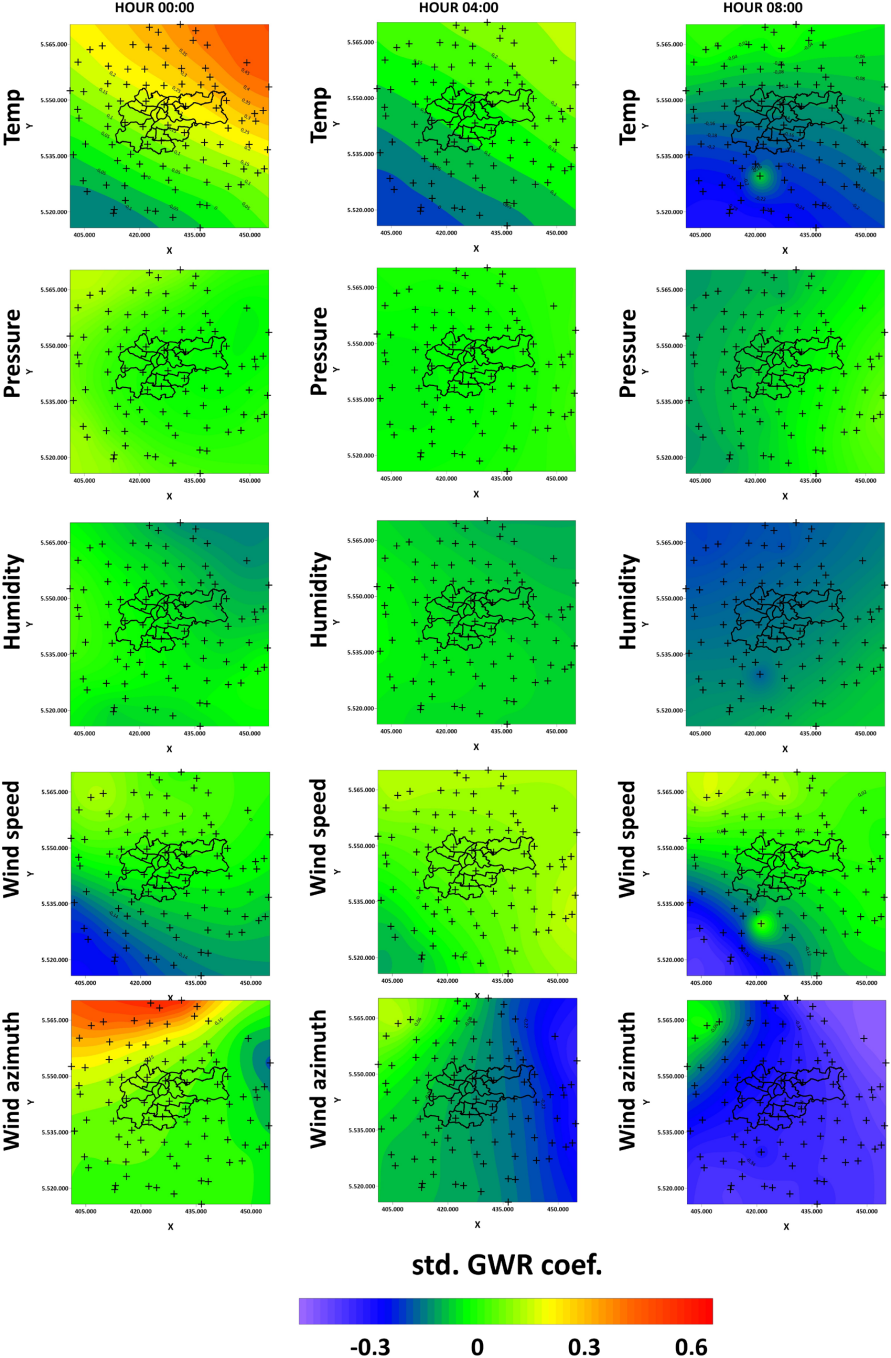
GWR coefficients for different meteorological factors on the 11th of March (outflow).

**Figure 11:**
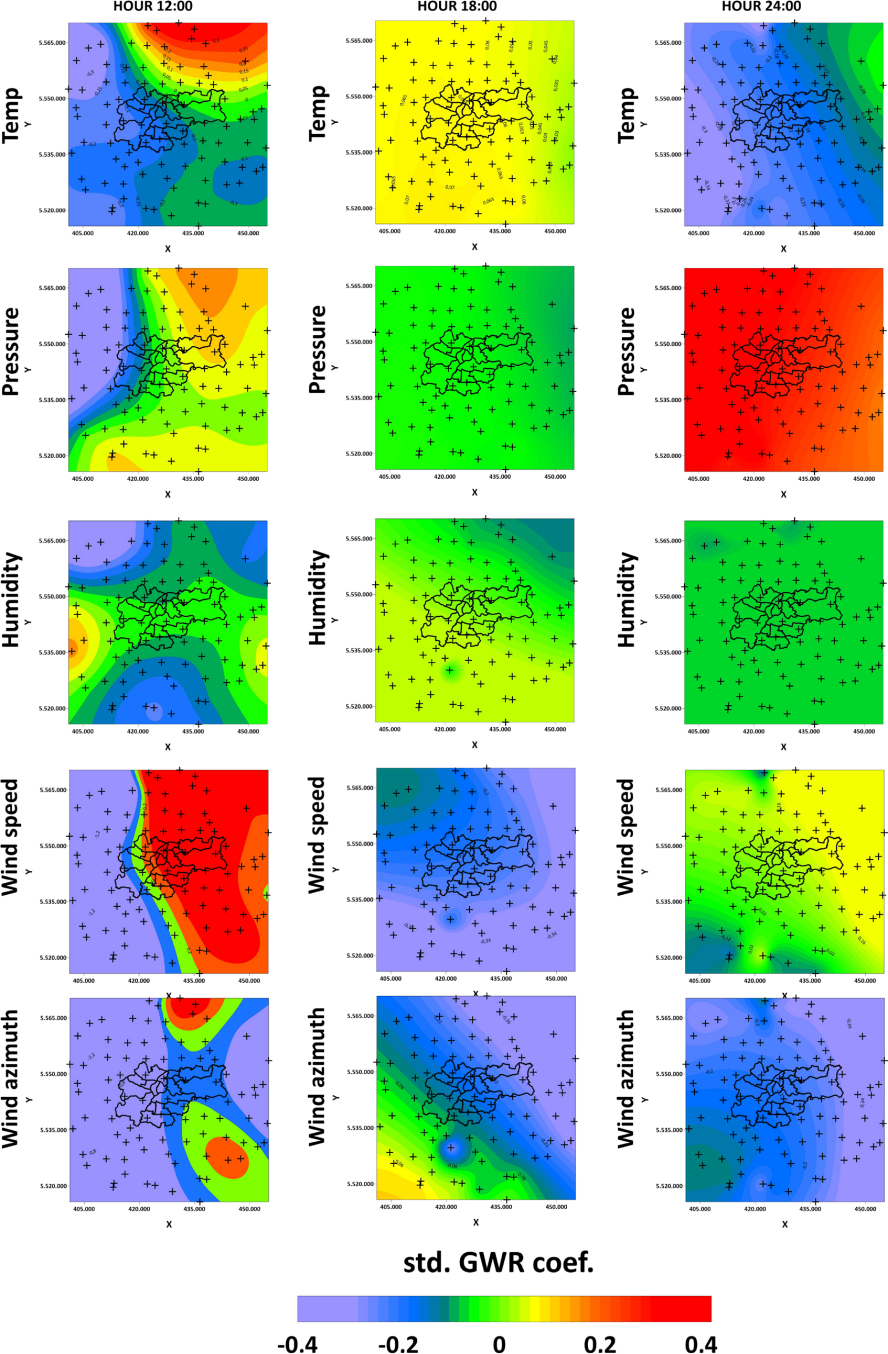
GWR coefficients for different meteorological factors on the 18th of March (inflow).

GWR of PM10 was performed on standardized data to assess the importance of individual meteorological factors. Figures 10 and 11 show GWR coefficients for different meteorological factors on the 11th and 18th of March: temperature, pressure, humidity, wind speed and wind azimuth. Tables 6 and 7 show the summary of the GWR models for 11th and 18th of March. The minimum and maximum values of GWR coefficients and standard errors for each factor as well as local R$^{2}$ is shown. The analysis shows that the factors which influenced PM10 concentration the most on 11th of March were temperature and wind speed and direction. The influence of temperature was most noticeable at 0:00, when the coefficient changed in the north-west-southeast directions in zones. In each of the three analyzed hours, the pressure and humidity effects were the least significant. Wind speed was most significant at 0:00 and 8:00. Wind azimuth had a very significant effect on PM10 values at each of the 3 h. At midnight, the highest positive coefficient values occurred northwest of Krakow. At 4:00, two zones of influence of wind azimuth on PM10 readings could be distinguished : low significance in the western part and high significance in the eastern part of the investigated area.

When the coefficients of the standardized GWR model on PM10 concentrations on March 18th are analyzed, a greater variation of values in the study area can be observed. The influence of these coefficients is also characterized by greater variability between observation hours. The influence of temperature was most significant at 12:00, when the highest positive values of the standardized coefficient occurred northeast of Krakow and the lowest negative values were northwest of Krakow. At 18:00, the influence of temperature was constant in the area, and at 24:00 the coefficient was negative and constant except for Nowe Brzesko and Proszowice in the north-east section, where the value of the coefficient was around 0. The pressure coefficients at 18:00 and 24:00 were constant in the whole area, whereas at 18:00 it was about 0, and at midnight it was high and positive (0.4). The greatest variation in pressure influence occurred at 12:00, with the largest positive values in the northeast and south, the most negative values in the west, and around zero in other regions. Humidity did not have a large effect on PM10 readings at 18:00 and 24:00, while it had a large effect on readings at 12:00. In the northeast, northwest, and south, the coefficient values were negative. At the easternmost and westernmost points, the values were positive, while in the rest of the area the coefficient was around 0. Wind speed and direction were significant in the model. At 12:00, the wind speed coefficients were the largest and positive in the east and smallest and negative in the west. At 18:00, the coefficient was negative across the area and had less spatial variation, while at 24:00 in the eastern part it was about 0.1, and in the rest of the region it was about 0. The wind azimuth coefficients at 12:00 show 2 anomalous areas of increased value. They cover places where sensors are located in Zreczyce, Jaroszowka, Winiary, Wiatowice and Slomniki, Prandocin. At 18:00, the values at the coefficient are zoned from positive values in the southwest to negative values in the northeast. At 24:00, there is the least spatial variation in the wind azimuth influence on the PM10 value.

**Table 6:** Summary statistics for GWR on the 11th of March.

	GWR coefficient		Standard error		Local R$^{2}$	
	Min	Max	Min	Max	Min	Max
Temperature	− 0.29153	0.481814	0.102446	0.161837	0.188082	0.521519
Pressure	− 0.11931	0.124524	0.110156	0.168907		
Humidity	− 0.20953	0.021871	0.10013	0.158674		
Wind speed	− 0.36667	0.159897	0.104612	0.258968		
Wind azimuth	− 0.46636	0.580548	0.106133	0.461677		

**Table 7:** Summary statistics for GWR on the 18th of March.

	GWR coefficient		Standard error		Local R$^{2}$	
	Min	Max	Min	Max	min	max
Temperature	− 0.38429	0.417453	0.105932	0.203544	0.128124	0.682986
Pressure	− 0.8018	0.396686	0.09397	0.169762		
Humidity	− 0.45138	0.179419	0.09713	0.204907		
Wind speed	− 2.54925	1.972361	0.120472	1.217837		
Wind azimuth	− 3.55514	0.759967	0.118913	1.473232		

**Figure 12:**
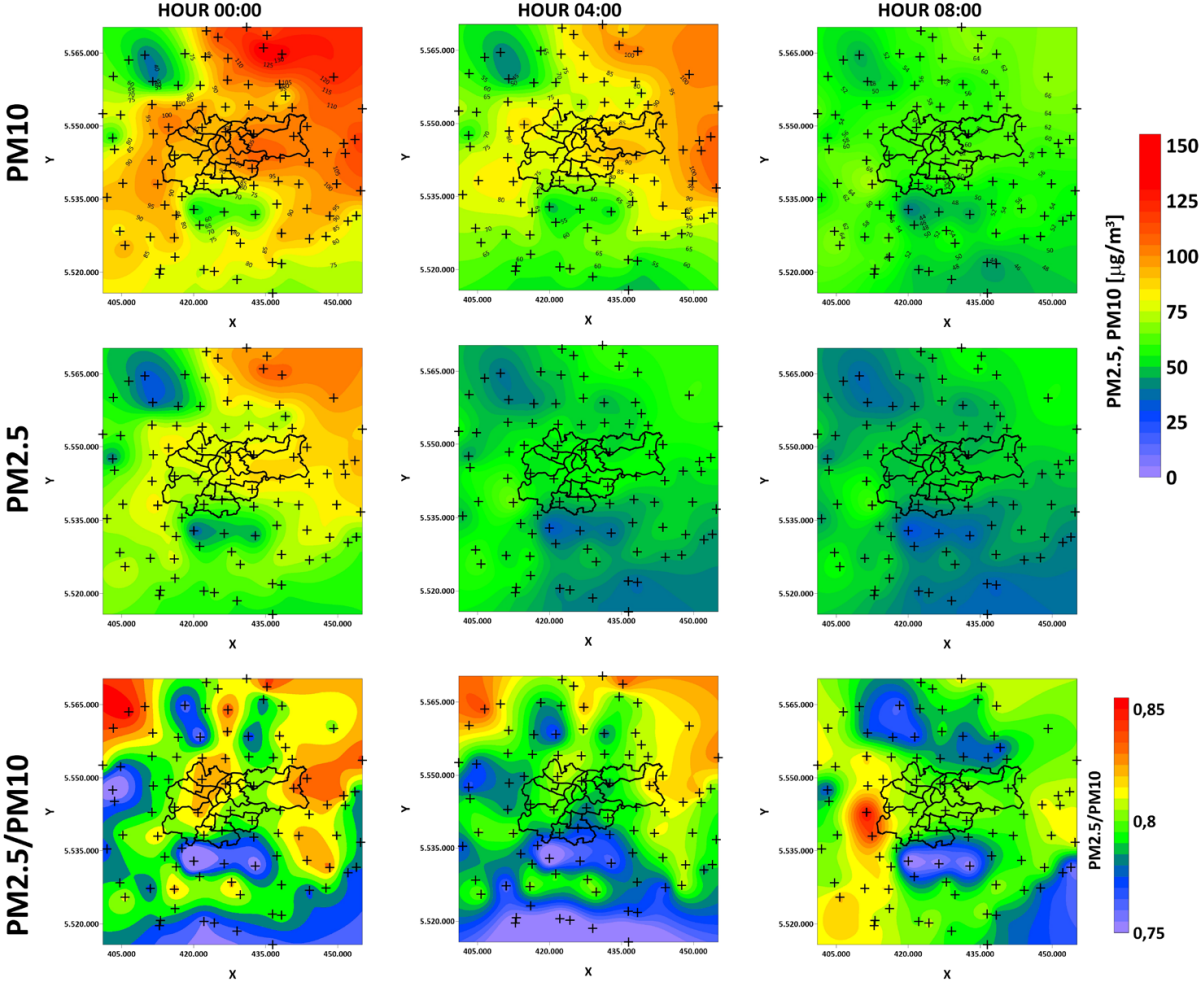
PM10 and PM2.5 GWR models with their ratio on the 11th of March (inflow).

**Figure 13:**
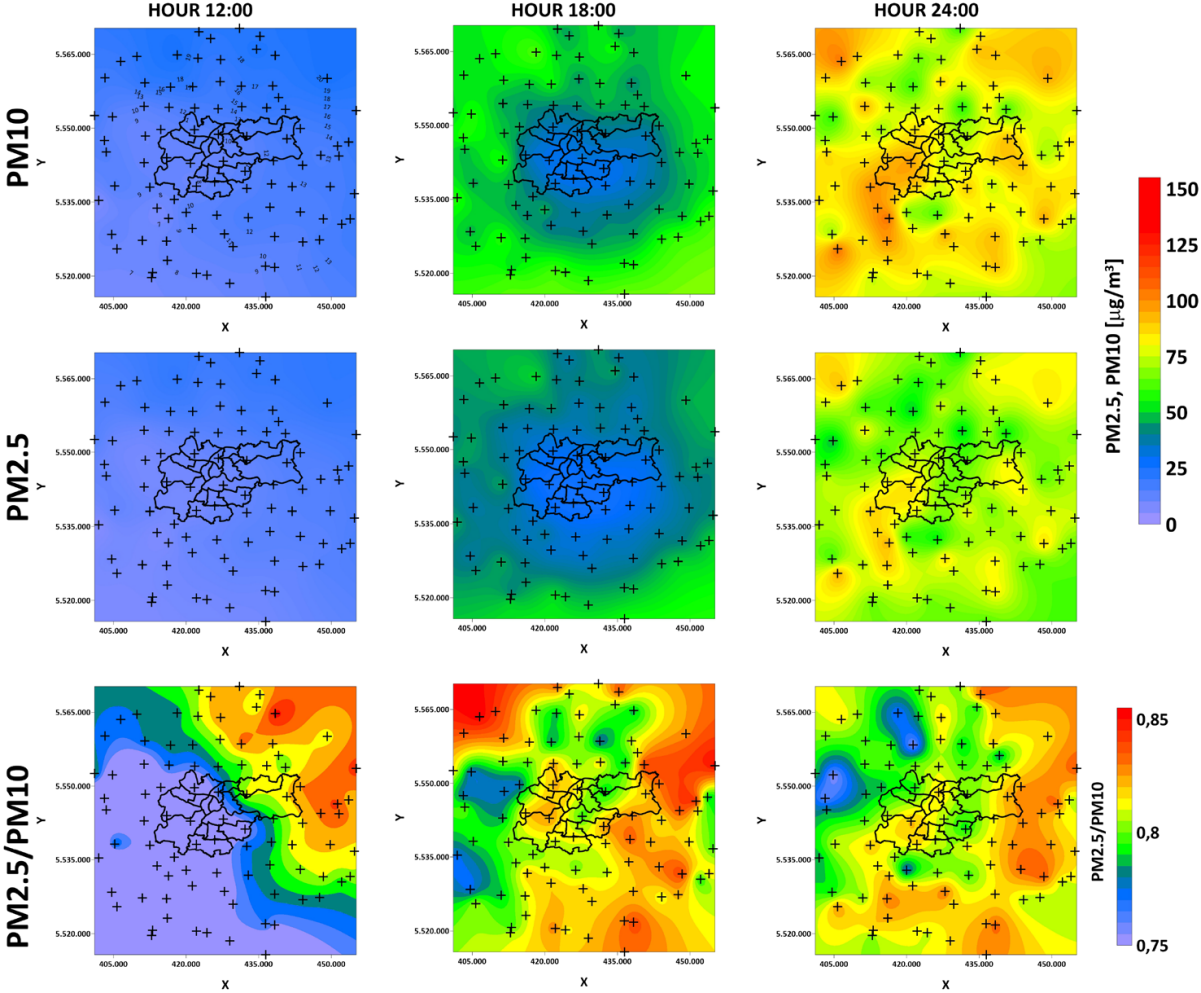
PM10 and PM2.5 GWR models with their ratio on the 18th of March (outflow).

Figures 12 and 13 show PM10 and PM2.5 GWR models with their ratios on the 11th and 18th of March. Previous research shows that PM10 and PM2.5 ratios are a good indicator of whether PM pollution is natural^51^ (mineral) or of anthropogenic origin^31^. The PM2.5/PM10 ratio can also be a useful tool for characterization of local atmospheric processes^30^. For both analyzed days, the ratios’ values are higher and start from 0.75. This allows the conclusion that, in the analyzed hours, the pollution in Krakow is of anthropogenic origin. The relative difference in individual hours seems to be interesting. Receivers located at a significant elevations in relation to the vicinity (Rzeszotary - SW12, Mogilany - SW13, Raciborsko - SE13) are characterized by almost constant ratio values (11th March - 0.75, 18th March - 0.8). The absolute values of PM10 and PM2.5 are also significantly lower there than at other LCS. The significantly higher values of the coefficients in the hours when pollutants are produced from combustion and during their migration coincide with the course of the main river valleys. PM outflow from Krakow on 11th March in the GWR models show the latitudinal system and the transport route of pollutants. PMs accumulate in the natural depression of the Vistula river valley, in which the city of Krakow is situated. Their outflow from the city is blocked from the north by the slope of the Ojcow and Krzeszowice plateaus, and from the south by the hills of the Krakow Upland and the Wieliczka Foothills. On March 18th, it is visible how the pollution bypasses the hills in the south of Krakow and is transported to the city through the valleys from the southwest.

## Discussion

EDA (Figs. 2, 3, 4 and 5) showed that the influence of particular meteorological factors on PM measurements is slightly different on 11th and 18th March. There are also some similarities. It is clearly visible that temperature had a direct impact on PM concentrations, especially the relative temperature perception below $0\,^{\circ }\mathrm{C}$ degrees. This is in line with the results of long-term analyses in the US^52^ and Poland^53^. This is also true for short-term temperature anomalies^12^. When people start feeling relative cold, the rapid emission of PMs from fossil fuel heating can be noticed. It is important to note that the humidity above 70% on both days is related to higher pollution concentrations. This local dependence may be related to the tendency for mists to form in Krakow during this period, which keeps pollution at the surface. Multi-annual research conducted in China shows similar conclusions: in urban areas increased pollution is related to fog^54^. Long-term observation of this factor for PM concentrations is recommended, including local climate of Krakow with warm and cold seasons.

The ambiguous correlation for pressure does not allow for a clear statement of whether its change has a positive or negative effect. Some studies show that this is the dominant factor^27^. In the short term, it may not vary significantly and will not dominate the solution. Wind speed is of great importance, as even a slight 1–2 m/s increase in speed strongly correlated with a decrease in pollution concentrations. This is because when particles start to move, the migration accelerates along the Vistula river valley. The south winds are associated with a lower concentration of pollutants. Less pollution in the case of southern winds is related to the presence of a natural terrain barrier on the southern side in the form of the Wieliczka Hills. The migration of pollutants in the case of the dominance of the west wind will be greater due to the Vistula valley. This is clearly visible in Figs. 12 and 13. Southern winds in this area may be associated with warm fen winds^55^, which is also important for pollution generated by household heating. Nevertheless, with such a low speed and relatively short observation time, it is difficult to talk about a noticeable trend for this factor. The multi-modal distributions show that, despite the small spatial area of the research that focus on Krakow and the nearest towns, both individual meteorological factors and concentration values vary greatly.

Spatial autocorrelation analysis of PM2.5 and PM10 values using local Moran’s *I* allowed us to separate clusters with high and low values as well as areas with anomalously high and low values in relation to neighboring areas. Clusters of high PM2.5 concentrations on both 11th and 18th March occurred mainly to the northeast and southwest of Krakow. Sensors with anomalously high PM2.5 concentrations on March 11th at 0:00 were Swoszowice and Nawojowa Gora, and Krzywaczka at 4:00. PM2.5 indications with increased values relative to neighboring sensors on March 18th were detected in Tenczynek (at 12:00), in Szczyglice and Wieliczka (at 18:00), and in Wieckowice (at 24:00). High-high clusters of PM10 indications were identified on March 11th at 0:00 at the same sensors as for PM2.5. At 4:00, high-value clusters of PM10 were determined in Wzgorza Krzeslawickie, Pietrzejowice, and Proszowice. On March 11th at 8:00, high-high clusters were identified in the south-west section in Brzeznica, Stanislaw Dolny, Brody, Zarzyce Male, and at one sensor to the north of Krakow in Prandocin. High-low outliers were detected on March 11th only at 0:00 (Nawojowa Gora) and at 4:00 (Krzywaczka). On March 18th, high-high clusters were recognized at 12:00 northeast of Krakow, at 18:00 in Gotkowice, and at 24:00 in Jeziorzany and Zarzyce Male. High-low outliers of PM10 were recognized at 12:00 in Tenczynek, at 18:00 in Szczyglice and Wieliczka, and at 24:00 in Wieckowice and Niepolomice. The locations of the high-low, high-high, low-high, and low-low PM10 and PM2.5 clusters are similar, but differences can be observed. High concentrations of PM2.5 in relation to the surroundings were observed in some places in the south of the city, where there are no significant clusters of PM10 concentrations. Two of the sensors are located in the vicinity of the main access roads to Krakow. The K9 Swoszowice sensor is located near the S7 expressway, while the SE17 Wieliczka sensor is located near the 94 expressway. Both are located near the A4 highway exits. Research shows that driving with studded tires in spring significantly increases the concentration of PMs^56^. The occurrence of abnormally high concentrations of PM2.5 in relation to neighboring clusters in the morning and evening hours may be associated with increased car traffic in these areas. This is in line with observations in Opole (Poland)^57^. The low-high clusters are mainly associated with land elevations, except for the K4 sensor located in the eastern part of Nowa Huta. This receiver is located in the wet area of Przylasek Rusiecki, near the eastern border of the city. It is also a part of strategic urban project called Krakow - Nowa Huta of the Future^58^. The low density of buildings and the proximity of forest complexes (the Niepolomicka Forest on one side of the river and the Przylasek Rusiecki complex on the other side) have a positive effect on air quality in this area. It was proved that urban composition has an impact on average PMs concentrations. Greater city fragmentation without densely built-up areas is positively correlated with lower PMs values^59^.

The hot-spots and cold- spots analysis using Getis–Ord $G_{i}^{*}$ statistics made it possible to identify a few sensors with increased PM2.5 and PM10 concentrations. Increased PM values were identified at single sensors located mainly outside Krakow. On March 11th, hot-spots were located in the northwest and west. This is in line with the highest values of pollutant concentrations and the dominant values of the GWR coefficients for temperature and humidity. Interestingly, it also clearly coincides with the river valley there and a significant terrain dip to the east of the Ojcowski and Krzeszowice plateaus. The two largest hot-spots on March 11 were at the NE17 and NE16 receivers (in Slomniki and Waganowice), which are located almost next to the river in the greatest depression and parallel to the river valley, where small elevations are present. This limits the possibility of the migration of pollutants accumulated in this depression. A completely different hot-spot location occured on March 18th. In the initial stage of observation (12:00), the hot-spots were located on hills, which is the opposite of what was observed on March 11th. The Ojcow Plateau region is also clearly distinguished when it comes to the values of temperature, humidity, and pressure coefficients for GWR. The combination of these meteorological factors probably made it necessary to start heating houses at 12 o’clock. This effect may not be always visible on distribution maps, because gridding may average the values of individual sensors based on the values at neighboring sensors. Hot-spot analysis is a useful tool to help make more accurate inferences than just studying pollutant distributions on maps. In general, the identified clusters provide good insight into the occurrence of local infrastructure and terrain. It can be seen that the clusters determined by local Moran’s index indicate highways, access roads and proximity to forests. Clusters determined by Getis–Ord $G_{i}^{*}$ statistics are mainly related to morphology, i.e., the occurrence of rivers and Ojcowski and Krzeszowice plateaus.

Performing a standardized GWR allowed us to analyze the influence of individual meteorological factors on PM10 indications. It is noticeable that the values of each coefficient change depend on the day and time of measurement. On the 11th of March, the greatest spatial variation in the influence of the coefficients in the model was for temperature, wind speed, and wind azimuth. It can also be seen that humidity had a significant influence in the Northeast region at 00:00, when high concentrations of PMs were observed there. You can see this effect in the KDE analyses, where, firstly, the concentration value increases with humidity, but secondly, in the highest range, a significant increase in the concentration of pollutants can be seen. The distributions of the temperature and humidity isolines are similar and are elongated in the north-west and south-east direction, while the influence of these factors is opposite. In places where the temperature has a positive effect, humidity has a negative effect. The overall influence of temperature is on average twice as large as that of humidity when the absolute values of the coefficients are taken into consideration. The wind azimuth and speed on March 11th had a big impact on the PM values, even though the wind was rather weak. This is an important observation that allows us to conclude that even a small amount of air movement improves air quality over time. The wind azimuth had a very big influence on the Ojcow plateau (comparable to temperature). In the next hours on that day, the concentration values decreased, as did the influence of meteorological factors on their values. The impact of wind varied according to the location of receivers and were different for those located on slopes and for those located in depressions in the terrain. Results are in line with the observations of Yang et al.^60^, which showed a large variability of individual meteorological factors depending on the measurement period, with wind being the dominant factor. On March 18th, there were much larger differences in the values of all analyzed coefficients. An influx of pollutants to Krakow could be observed. The greatest spatial variation was observed at 12:00 for each of the meteorological factors: temperature, pressure, humidity, wind speed and wind azimuth. At that time, the pollutant concentrations were very low and were within European standards. The longitudinal distribution of meteorological factors such as temperature, pressure, wind speed, and direction is visible. It coincides with the division of the city at this hour into the eastern and western parts in terms of the value of the PM2.5/PM10 ratio. On this basis, it can be concluded that the meteorological factors favored the concentration of pollutants from the morning road traffic peak in the eastern part of the analyzed region, while in the western part of the city the factor derived from fuel combustion was not dominant, but only secondary anthropogenic dust was present (for more information on the PM2.5/PM10 analysis, see the last paragraph of this section). At 18:00, the most influential coefficients were wind speed and wind azimuth. Again, there is a strong coincidence between the coefficient values for wind parameters and the terrain. The region can be divided into the Ojcow plateau and its slope, which reaches as far as the Vistula valley, and the southern part below the Wieliczka Uplands. At 24:00 the highest standardized coefficient and constant for the whole area was the pressure coefficient. Temperature and wind azimuth were also important in that model. The distribution of isolines for temperature and wind speed is similar and again shows similarity to the PM2.5/PM10 ratio distribution map. These factors can affect the relative feeling of cold and act as triggers for household heating with solid fuels and, in consequence, the production of PMs from combustion. Temperature drop increases the need for the fossil fuels combustion^61^. For pollution outflow, in the absence of thermal inversion, the wind azimuth is the dominant factor. Relatively low wind speed is enough. The pressure did not change significantly and its influence was close to zero at each analyzed hour. The situation is different in the case of the inflow of pollutants. In the initial phase, the dominant factor is wind speed and direction. Pressure plays a dominant role in preventing the movement of pollution to the city. The share of temperature can be indirectly analyzed to indicate PM emission sources.

Some studies have shown that higher PM2.5/PM10 ratio values (about 0.9) are associated with anthropogenic processes such as fuel combustion (by heating houses or in car engines), and lower but still high values (about 0.7) are related to other anthropogenic factors like mining, secondary dust lifting by car or bicycle wheels, and agriculture^62^. This relationship is clearly visible in the analyzed hours on March 11th and 18th. In places where the increased emission of pollutants from the combustion of solid fuels occurred, an increase in the ratio was observed. It can be seen that the dominant factor for the migration of pollutants is related to the valleys that coincide with the main rivers in the analyzed region. In elevated regions, PM2.5/PM10 ratio values remain at levels characteristic of anthropogenic non-fuel emissions (around 0.7-0.8), even if combustion has occurred there. Based on the analysis of the PM2.5/PM10 ratio, the influence of the unfavorable geographical situation of the city is clearly visible. Pollution related to the combustion of solid fuels accumulates in the city even though household heating with solid fuels is forbidden there. Municipalities located on the northern and southern elevations are less exposed to long-term exposure, even if they are the main emitters of these pollutants. Of course, this situation may vary in scale depending on the meteorological situation, including phenomena such as temperature inversion^63^.

## Conclusion

The problem of air pollution is important for public health. The impact of individual meteorological factors on the concentration of PMs and the impact of macro-geographical factors on their migration has been analyzed in many studies whose main focus was finding long-term relationships based on sparse-sensor grids. The subject of these studies is a short-term spatial analysis based on a dense and regularly sampled network of 100 LCS receivers whose measurements are characterized by relatively high uncertainty. The use of dedicated machine learning techniques by data providers allowed for compliance with the reference stations at the level of 99%. The research was conducted in the early spring during the COVID-19 pandemic. This allowed for the observation of pollutants mainly from the combustion of solid fuels without the additional background pollution resulting from car transportation.

The use of geostatistical methods made it possible to accurately trace places with increased or decreased PM emissions and the location of places that are anomalous in relation to their surroundings.To determine the share of individual meteorological factors, GWR was used based on standardized data. This allowed the performing of a quantitative time-space analysis of individual variables using a common scale while preserving differences in their ranges. The analysis of PM2.5/PM10 ratio values made it possible to distinguish between pollutants generated from combustion and other anthropogenic sources. The high usefulness of this indicator has been demonstrated for tracking solid fuel heating sources. These sources were located outside Krakow. Analysis of the influence of meteorological factors on the concentration of PM air pollutants is a difficult and ambiguous task. The influence of individual meteorological factors, depending on their combination with other factors, on one day gave the opposite dependence than on another study day. The roles of these factors depend on whether the outflow or inflow is analyzed. For the spring period in this terrain regime, the biggest impact on PM outflow was wind azimuth (west and north-west), while the least relevant was pressure. For inflow, the most important factors in the initial phase were wind speed and direction. Later, air pressure was the dominant factor in terms of trapping pollutants in the city. Terrain plays a very important role in the production and migration of pollutants. On the studied days, pollution accumulated along the river valleys. Krakow, which located in the Vistula valley and is limited to the north and south by hills, has a very unfavorable location which favors the accumulation of external pollution. Longitudinal winds have bigger impact on both the inflow and outflow of PMs than winds from perpendicular directions. This conclusion cannot be directly transferred to other cities without detailed investigation of local terrain.

Our study show how complicated it is to combine many factors into a single cause-effect sequence. Determining the general relationships is not as complicated as trying to describe them hour by hour, when significant PM concentration changes can occur. The presented statistical analysis and its results may, in the future, be used as a data source for continuous analysis of time series with the use of machine learning and artificial intelligence. This research shows that for air pollution management planning, a localized multi-factor impact study should be performed.

## Data Availability

Publicly available datasets from Airly sensors were analyzed in this study and can be found here: (https://map.airly.org/, accessed on 17 Feb 2022). API documentation from Airly is available here: (https://developer.airly.org/en/docs, accessed on 17 Feb 2021). Publicly available datasets from the Chief Inspectorate For Environmental Protection database were analyzed in this study. This data can be found here: (http://powietrze.gios.gov.pl/pjp/home, accessed on 17 Feb 2022). API documentation is available here: (http://powietrze.gios.gov.pl/pjp/content/api, accessed on 17 Feb 2022).
